# A Clinical Psychological View about Delusional Characterizations in Subjects with Schizophrenia Spectrum Disorder during the COVID-19 Period

**DOI:** 10.3390/jcm12072698

**Published:** 2023-04-04

**Authors:** Marilena Maglia, Maria Salvina Signorelli, Antonino Petralia, Idria Verduzzo, Concerto Carmen, Alessandro Rodolico, Jennifer Di Piazza, Pasquale Caponnetto

**Affiliations:** 1 Department of Educational Sciences, Section of Psychology, University of Catania, 95121 Catania, Italy; 2 Center of Excellence for the Acceleration of Harm Reduction (COEHAR), University of Catania, 95121 Catania, Italy; 3 Department of Clinical and Experimental Medicine, University of Catania, 95121 Catania, Italy; 4 Hunter Bellevue School of Nursing, Hunter College, City University of New York, New York, NY 10065, USA

**Keywords:** schizophrenia and COVID-19, schizophrenia delusions during COVID-19, schizophrenia delirium and COVID-19, characterizations of delusions during COVID-19, psychosis and COVID-19

## Abstract

(Background) The period experienced during the COVID-19 virus and the respective social regulations associated with it caused enormous psychosocial stress. (Objective) The objective of the present work was to observe whether the lived period induced a change in delusional characterizations in subjects with schizophrenia spectrum disorder. (Methods) A systematic literature review was conducted following the PRISMA 2020 guidelines for systematic reviews of the PRISMA GROUP. The literature search was conducted from November 2021 to May 2022, using various scientific platforms including PubMed. (Results) A total of 865 articles were found, from which 176 duplicates were removed. The remaining articles were reviewed by reading the titles and abstracts; fourteen were included. (Conclusions) During this research, it was possible to confirm the initial thesis, namely that delirium absorbs external reality by being modified by it. It was observed that the speed of absorption is estimated to be directly proportional to the speed of the modification of social reality and to the impact that the latter has on the subject’s private sphere. Moreover, the situation of radical change represented a condition of abnormal psychosocial stress, which led to an increase in diagnoses of schizophrenia spectrum disorders and, specifically, a weighty increase in diagnoses of brief psychotic disorder (BDP). In the coming years, it is estimated that there will be an increase in diagnoses of schizophrenia spectrum disorder caused by both environmental and biological factors.

## 1. Introduction

Schizophrenia is generally divided into three groups of symptoms: positive, negative and cognitive.

Positive symptoms are typically represented by psychotic behavior not evident in healthy individuals. Generally, individuals with positive symptoms of schizophrenia lose touch with reality.

Negative symptoms often lead to poor motivation and a reduction in intentionality and goals. 

Cognitive alterations (attention, sense of orientation, memory) are usually detected before the onset of real psychotic symptoms, providing us with a clinical picture that gives us the first alarm bells.

The term delusion refers to the presence of strong erroneous, pathological beliefs that cause an alteration in the subject’s sense of reality. Delusions take root despite the evidence that may be present in reality. Depending on their content, they are subdivided into religious, delusions of grandeur, persecution, reference, somatic, etc. For instance, in the persecution delusion, the subject may mistakenly believe that he is being followed constantly or that there is a conspiracy against him. Delusions often occur in comorbidity with hallucinations, which give the delirium even more value and belief, and represent one of the most intrusive symptoms of the disorder, as they alter the patient’s sense of reality.

In the Diagnostic and Statistical Manual of mental disorders (DSM-5) [1] schizophrenia spectrum disorders include schizophrenia, schizophreniform disorder, brief psychotic disorder, schizoaffective disorder and delusional disorder. 

Additionally included in DSM-5 in Section 3, with conditions requiring further study, is the attenuated psychosis syndrome, which has not yet been included in DSM-5 as it focuses more on the future risk of onset of the disease and not on the subject’s present mental condition.

Schizophrenia spectrum disorders, like most mental disorders, are caused by the intertwining of an etiological basis and an environmental system; there must be the joint presence of both factors. Our research focus is on a circumscribed historical period: the period characterized by the presence of the COVID-19 virus in our social context. The presence of this virus led to a global pandemic, which is still ongoing.

The virus initially spread in Wuhan, China, in December 2019, and subsequently, in a few months, throughout the rest of the world, leading to the introduction of several regulations aimed at preventing the spread of the virus, including constant hand washing; the use of a protective mask both outdoors and indoors; social distancing of at least one meter; and the use of protective gloves. In addition, in the event of a positive COVID-19 test, patient self-isolation was implemented. The most stressful phenomenon occurred with the institution of the total closure period, which lasted an average of three months. During this period, the population was confined within their homes, which they could only leave for extreme, self-certified needs. This led to a change in social reality and the emergence of coronaphobia (fear associated with COVID-19) [2]. 

The aim of the present work is to observe, through a systematic review of the literature, whether the period experienced, characterized by the presence of the Coronavirus Disease 19 (COVID-19), influenced the delusional characterizations in subjects with schizophrenia spectrum disorder and was a predictive factor in the development of the disorder.

Several specialists call this period one of the most traumatic events of recent decades due to its peculiarities, including the indiscriminate mortality even among the young and the preventive measures that were applied. There was a radical change at a social and relational level, caused by the regulations introduced to stop the spread of COVID-19. The starting hypothesis was that the period experienced, characterized by the presence of the current regulations linked to the dissemination of COVID-19 and the overabundance of media information, induced a modification in the pathoplasticity of the delirium in subjects with schizophrenia spectrum disorder. Thus, it was expected that this period, a source of enormous stress, would lead to a modification within the delusions, as delusions tend to absorb external reality. Furthermore, we hypothesized an increase in diagnoses of schizophrenia spectrum disorder caused by COVID-19.

This research confirmed the initial thesis by highlighting the following points: That delirium, as has also been observed in the past, absorbs external reality and is modified by it. Indeed, during this systematic literature search, it was observed that changes in the delusional content occurred in connection with social changes because the delusional content is always intertwined with the cultural and social reality and the everyday life of the subject, so it follows that every external variation has an internal impact on it. The central themes of the delusions generally remain unchanged but the characterizations within them change. In support of this, several clinical cases were observed within the research, highlighting the changes that had occurred in the pathophysiology of the delusions, which, especially during the initial weeks of the pandemic, centered on the COVID-19 virus [3].

The situation of a radical change in reality that one found oneself experiencing represented a condition of abnormal psychosocial stress, which led to an increase in diagnoses of schizophrenia spectrum disorders and, specifically, a weighty increase in diagnoses of brief psychotic disorder [4].

Furthermore, it has been shown that an overabundance of information (a phenomenon termed infodemia) caused by the mass media positively correlates with the development of conspiratorial thinking and the onset of delusions [5].

Several studies have also shown that individuals diagnosed with schizophrenia spectrum disorder did not experience symptom exacerbations during the period of social isolation, probably due to the inherent characteristics of the disorder [6]. In the coming years, diagnoses of schizophrenia spectrum disorder are expected to increase due to both environmental and biological factors, including exposure to the virus [7]. The main aim of this research work is to identify how the delusional characterizations, in response to perceived stress, are differentiated in subjects with schizophrenia spectrum disorder. It also wants to observe how the delusions have changed on the basis of changes in the social context and also to identify how the period experienced, being a source of psychic stress, has led to an increase in diagnoses of schizophrenia spectrum disorder.

## 2. Materials and Methods

### 2.1. Objective of the Work

The main aim of the present research work is to identify how the delusional characterizations, in response to perceived stress, are differentiated in subjects with schizophrenia spectrum disorder. It also wants to observe how the delusions have changed on the basis of changes in the social context and also to identify how the period experienced, being a source of psychological stress, has led to an increase in diagnoses of schizophrenia spectrum disorder.

### 2.2. Research Strategy

A systematic review of the literature on the topic was conducted following the PRISMA 2020 guidelines for systematic reviews of the PRISMA Group. The literature search was conducted from November 2021 until March 2022, using the PubMed platform, by entering the following keywords: schizophrenia and COVID-19, schizophrenia delusions during COVID-19, schizophrenia delirium and COVID-19, characterizations of delusions during COVID-19, psychosis and COVID-19. 

### 2.3. Eligibility Criteria

All articles written in the English language published from 2020 to 2021 that met the following criteria were included in the search:

Participants: patients with schizophrenia spectrum disorder (premorbid, prodromal, central and late phase).

Intervention: Pharmacological intervention used in order to restore the sense of reality of the subject with the psychotic manifestation related to the illness. A psychotherapeutic intervention was then applied in order to restore the patients’ psychosocial functions.

Comparison: It was examined how the COVID-19 period induced changes in delirium pathobiology in subjects with schizophrenia spectrum disorder.

Outcome: There was an observation of COVID-19-related delusional characterizations in subjects with schizophrenia spectrum disorder. An increase in new-onset diagnoses was observed, e.g., an increase in diagnoses of brief psychotic disorder.

### 2.4. Data Extraction

The data were extracted using a format that provided, for each article, the author, year, title, country where the research was conducted, type of study conducted, measures used and results.

### 2.5. Search Results

**Figure 1:**
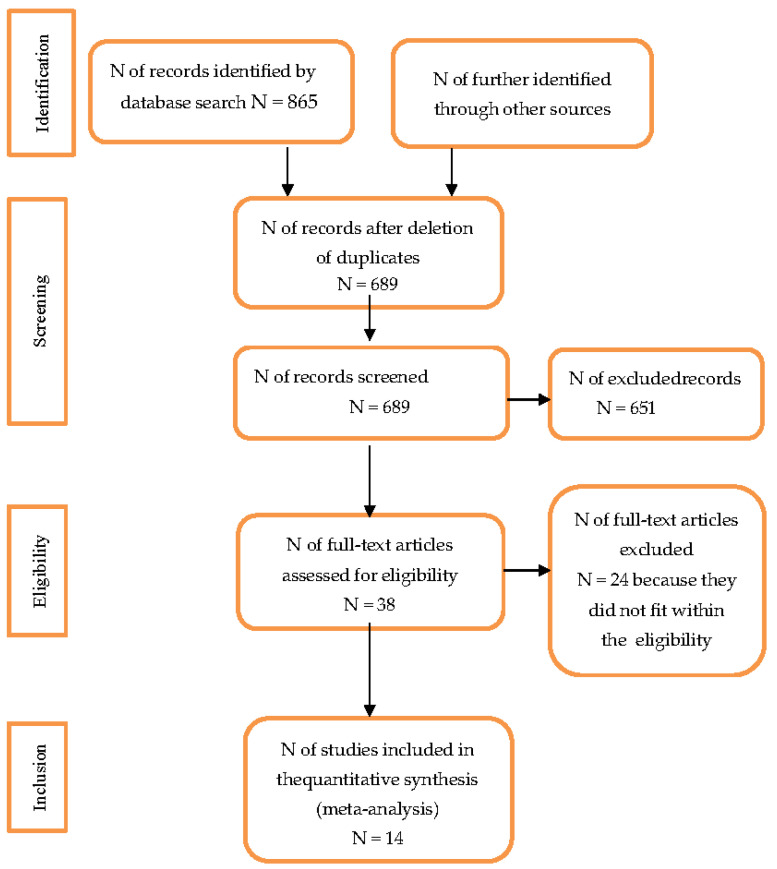
PRISMA 2020 flow diagram.

Initially, 865 articles were found using the PubMed platform. In total, 176 duplicates were eliminated. The remaining 689 articles were examined by reading the titles and abstracts and 651 were excluded because they did not meet the inclusion criteria, as they analyzed the subject matter through a specific medical approach and did not focus on our objective of analysis. The remaining 38 articles were examined in full and 14 were included. The inclusion criteria were participants with a diagnosis of schizophrenia spectrum disorder, psychotherapeutic and pharmacological intervention, and delusions centered on and emphasized by COVID-19. The exclusion criteria were analysis of the topic from a medical or health point of view, articles that did not deal with the importance of COVID-19 in the symptom manifestation, articles where the sample did not present the diagnosis of schizophrenia spectrum disorder (premorbid, prodromal, central, late phase). The data obtained show that this topic is unexplored. The above description is summarized in the flowchart in Figure 1.

### 2.6. Bias Risk Assessment

The risk of bias for the included studies was assessed with the Cochrane risk of bias tool for randomized trials, version 2 (RoB 2). The control analysis with the RoB 2 is summarized in Table 1.

### 2.7. Cochrane Risk of Bias Tool for Randomized Trials, Version 2 (RoB 2)

The RoB 2 tool was developed to assess the risk of bias in randomized controlled trials (RCTs). RoB 2 assesses the risk of bias in five key areas of an RCT:

1. Randomization: the proper execution of randomization of the sample of study participants, ensuring that it is random and that there is no manipulation of any kind.
2. Assignment: the proper execution of assigning study participants to treatment and control groups, ensuring that it is random and that there is no manipulation of any kind.
3. Interventions: the proper execution of the treatment and control interventions, ensuring that they are applied uniformly to the participant groups and that there is no manipulation of any kind.
4. Lack of data: the proper handling of missing data, ensuring that there is no bias in the study results due to missing data.
5. Results: the proper execution of the evaluation of results, ensuring that there is no bias in the interpretation of the study results.

Each area is assessed according to a set of questions that can be answered with “low risk,” “uncertain risk,” or “high risk.” The overall bias risk assessment is then expressed as “low risk,” “moderate risk,” or “high risk” for each area and for the entire study.

Assessment of risk of bias with the RoB 2 tool is important to help ensure that clinical trial results are reliable and can be used safely to make informed clinical decisions.

## 3. Results

The studies included highlighted how this period led to a mutation in the pathoplasticity of delusions and how there was a weighty increase in diagnoses of schizophrenia spectrum disorders, specifically brief psychotic disorder (BDP). Several studies have shown that there is a link between pandemics and the onset of psychosis, which has already been highlighted in the past. The data extraction of these studies can be visualized in Table 2.

### 3.1. Does the COVID-19 Pandemic Affect the Delusional Content of Patients with Psychosis? An Observational, Retrospective, Cross-Sectional Study

The aim of the research conducted by Pérez-Balaguer et al. (2021) was to observe the impact of the COVID-19 pandemic on the mental health and delusional content of patients who visited the HUB of a hospital in Madrid [8]. The results showed that COVID-19 was incorporated into delusions and induced the onset of psychotic symptoms. During the first month, COVID-19 influenced the delusional content of 38.5% of the hospitalized patients and was the direct trigger in 46.2% of the cases. During the second week, it was a determining factor in both cases in 100% of the recorded cases. A decrease was observed thereafter.

### 3.2. Paranoia, Hallucinations and Compulsive Buying during the First Phase of COVID-19 Outbreak in the UK: A Preliminary Trial Study

This study, conducted by Lopes et al. (2020) [9], had the main objective of examining the impact that COVID-19 had on psychotic symptoms during the first phase of the pandemic. Several variables were examined, including self-isolation, COVID-19 symptomatology and exposure to virus-related news. The sample subjects were divided into two conditions. In the experimental condition (*n* = 185), subjects were exposed to COVID-19-virus-related news, unlike subjects in the control group (*n* = 176).The following instruments were used: the Trust in Politicians and Trust in the Political System Scales [19], the Lubben Social Network Scale [20], The Fear of COVID-19 Scale [21], the Paranoia Checklist [22], Launay and Slade’s Predisposition to Hallucinations Scale [23] and the Compulsive Buying Behaviour Scale [24]. The research results highlight how students and the employed were more prone to paranoia and the development of psychotic symptoms, and how isolation conditions were a strong psychosocial stressor. It was also observed that subjects in the experimental group had a statistically significant increase in feelings of political distrust, paranoia and hallucinatory experiences, highlighting how exposure to conspiracy theories and information overload induced the development of delusional thoughts related to the pandemic.

### 3.3. Clinical Characterization of Short Psychotic Disorders Triggered by the COVID-19 Pandemic: A Multicenter Observational Study

The aim of this two-month study by Valdés et al. (2021) [10] was to observe the clinical profile of subjects diagnosed with BDP triggered by COVID-19. Subjects between 18 and 65 years of age with a diagnosis of BDP, who visited hospitals in Andalusia, were included. None of the subjects in the sample were positive for the COVID-19 virus. Initially, 57 subjects were identified, of which 33 were included who met the DSM-5 diagnostic criteria of BDP triggered by COVID-19. Half of the subjects manifested symptoms of first-order schizophrenia and a quarter of the sample showed suicidal symptoms. The remarkable fact, in addition to the increase in BDP diagnoses caused by the pandemic, is the observation that, in more than half of the cases (57.6%), the delusional content was related to COVID-19.

### 3.4. Short Psychotic Disorder during the National Lockdown in Italy: An Emerging Clinical Phenomenon of the Coronavirus Pandemic

The aim of this research conducted by D’Agostino et al. (2020) [11] was to examine the impact of the COVID-19-related social situation on the population. Various clinical cases of in-patients with a diagnosis of brief psychotic disorder were reported. The following instruments were used: the Brief, the Psychiatric Rating Scale, the Paykel interview and the SCID-II. The cause of the symptoms was found to be the strong stress associated with the social situation characterized by the presence of COVID-19. In addition, the subjects underwent an antigenic swab in order to exclude the presence of the COVID-19 virus.

### 3.5. Peripatetic Psychiatric Emergencies: Impact of the COVID-19 Pandemic on Patients by Second Diagnostic Subgroup

In this research conducted by Seifert et al. (2021) [12], information was collected on patients over the age of 18 years, examining a total of 75 patients. Analyses showed that eight patients presented with delusions and hallucinations centered on COVID-19 and five stated that the social situation and the loss of work represented a condition of enormous perceived psychosocial stress.

### 3.6. Has Psychopathology in Indian Psychiatric Patients Changed Following the COVID-19 Pandemic?

In this research, conducted by Behere et al. (2020) [13], several clinical cases of patients who visited the Wardha hospital were examined. It was observed how the pandemic induced the development of psychosis and how the content of the delusions incorporated COVID-19.

### 3.7. Psychosis in Quarantine Patients Related to COVID-19: A Case Series

This research describes three clinical cases of patients admitted to the University Hospital of Padua between 20 and 25 March 2020.The subjects had no psychiatric history. The onset of psychotic symptoms was found to be related to quarantine [14].

### 3.8. Brief Psychotic Disorder during the COVID-19 Pandemic: A Case Series

In this study, three clinical cases of patients admitted due to the onset of psychotic symptoms are examined; the subjects were diagnosed with BPD [15]. The admissions occurred within less than one month of the initial national blockade. It was revealed that somatic and auditory delusions are linked to COVID-19, which was incorporated into the pathology of delirium. It was observed that one of the predictors of the onset of psychotic disorders, during quarantine, is living alone [4].

### 3.9. Short Psychotic Disorder Associated with COVID-19

Three clinical cases, identified by Ferrando et al. (2020) [25], of COVID-19-infected patients who experienced new-onset psychosis treated with antipsychotics are described in this study. Two of these patients had a psychiatric history behind them. This study highlights how the virus attacking brain areas can induce the development of psychiatric symptoms. It also highlights the correlation between psychosis and viruses already identified by Menninger in 1918.

### 3.10. Psychosis and Isolation Infodemia Resulting in the First Hospitalization during the COVID-19 Pandemic: A Case Series

Three clinical cases of subjects with BPD are described, analyzing how forced isolation is a condition of strong perceived stress and how the latter can induce the development of psychosis. Furthermore, it is highlighted how infodemia is a predictor of the development of delusions and how the content of these delusions was centered on COVID-19 [16].

### 3.11. Psychosis in a Patient with COVID-19-Related Anxiety: A Case Report

This article reports a clinical case of a woman with no psychiatric history who presented with psychotic symptoms caused by persistent anxiety towards COVID-19. Furthermore, the “Coronophobia” identified by Asmundson and Taylor (2020) is examined, who highlight the relationship between persistent anxiety and the development of psychotic symptoms [2].

### 3.12. COVID-19 Paranoia in a Patient with Schizophrenic Psychosis: A Case Report 

Clinical case of a subject who, due to enormous perceived stress, manifested delusional symptoms [3].

### 3.13. Blockage and Psychosis: A Paranoid Delusion

In this study by Nava et al. (2020) [17], a clinical case is examined. This is the first case of paranoid delirium (according to DSM-5 criteria) caused by lockdown and misinformation in an uninfected COVID-19 patient admitted to the psychiatric department. Lockdown and misinformation can cause the development of a wide and nonspecific range of psychiatric symptoms. In this patient, psychotic symptoms were preceded by a depressive state, anxiety, and insomnia.

### 3.14. Fear of Spreading COVID-19 Infection in a Woman with Psychotic Illness Leading to Suicide and Homicide Attempts: A Case Report

In this article, a clinical case highlights how the fear of contracting and spreading the COVID-19 virus led to suicide and homicide attempts in order to avoid the associated social stigma [18].

## 4. Discussion

Research conducted on the subject has shown how the period experienced, characterized by the presence of the COVID-19 virus, has led to changes in the pathology of delusions in subjects with schizophrenia spectrum disorder, observing how changes in the delusional content occurred in relation to social changes. The delusional content is always intertwined with the cultural and social reality and the daily life of the subject, so it follows that any external variation has internal repercussions.

The central themes of the delusions remain unchanged, but the internal characterizations change. It is unclear how quickly the delusions absorb social changes, but it is estimated that the speed of change is directly proportional to the extent of the change and its impact on the subject’s private sphere. The following is an example of a clinical case of a patient with schizophrenic spectrum disorder [3]. The subject, brought to the emergency room by his father, is a 43-year-old unmarried man. For about a week, the patient claimed to hear voices accusing him of neglectful acts of family care towards his parents. The voices accused him of having contracted COVID-19 and of being the cause of spreading the virus to his family and neighbors. In addition, he claimed to be immune to the virus as he had previously been infected by a Chinese message received on WhatsApp (a manifestation of the initial stigma towards the Chinese people from which the spread of COVID-19 originated). At the time of his admission, he presented with auditory hallucinations, high tension, anxiety and depressed mood, but appeared relatively consistent with external reality. Back in 2011 and 2019, the patient had been admitted for similar delusional symptoms, treated with the administration of an antipsychotic, and had been diagnosed with acute polymorphic psychotic disorder and paranoid psychosis. In this clinical case, it is evident how the social period and related fears were incorporated into the patient’s paranoid delirium very quickly and led to an exacerbation of the symptoms [26]. It was also possible to observe that there was an increase in diagnoses of schizophrenia spectrum disorder, specifically a surge in diagnoses of brief psychotic disorder. One of the examined cases of BPD diagnosis is that of a 31-year-old unmarried man who worked as a courier before the pandemic. He had no psychiatric or physical history. The first symptoms began with the loss of his job due to the infrastructure blockade. The economic worries induced persistent feelings of anxiety and sleep–wake cycle disturbances in the subject. In this highly tense state, he developed persecution mania centered on COVID-19. Taken to the emergency room, he was hospitalized for about three weeks with a diagnosis of brief psychotic disorder, according to DSM-5 diagnostic criteria. As can be well observed, the pandemic period was a factor of strong perceived psychosocial stress that was one of the predictors of the development of schizophrenic spectrum disorders. These stressogenic conditions were generated by the perceived enormous stress caused by the radical change in society in just a few days, aimed at containing the spread of the COVID-19 virus. This perception of psychosocial stress [4] was accentuated, as evidenced by several research studies conducted by Hu by the frequent media reports on COVID-19 leading to infodemia (overcrowding with inconsistent information). This has led to a strong stigma, anticipated guilt and intense fear around COVID-19 [27]. This fear, termed “Coronaphobia”, stems from the dread of contracting and spreading the virus and is associated with the manifestation of psychotic symptoms in patients with a psychiatric history [2]. This social framework of uncertainty and fear correlated positively with the spread of conspiratorial thinking, as observed during the lockdown in America where there was a high peak of conspiracy theories explaining the spread of the virus in various ways [28]. In support of this, in a study conducted in Jordan by Sallam between 11 and 14 April 2020 [26], it was found that out of a total sample of 3150 subjects, 47.9% said they believed that the COVID-19 pandemic was the result of a global conspiracy.

In the specific case of conspiracy theories, as the income or educational qualification variable increases, the tendency of subjects to adhere to popular conspiracy thoughts decreases. In a research conducted in Spain by Pérez-Balaguer et al. (2021), there was an opportunity to confirm our research hypothesis that a period of living with the COVID-19 virus led to a change in the pathology of delusions and an increase in diagnoses of schizophrenia spectrum disorder [8]. Specifically, during this research, patients visiting the emergency room and the UHB of a tertiary hospital in Madrid during the first three months following the initial country blockade were analyzed. The research was conducted between 11 March and 11 June 2020. A total of 414 subjects were observed, older than 18 years of age, within the research out of an initial total of 431. The study was conducted through the consultation of medical records by two professionals. Several variables were taken into account, including age, gender, income, voluntary or involuntary consultation, socio-demographic variables, area of hospitalization, presence of psychotic symptoms related to the COVID-19 pandemic, the motivation for consultation and the presence or absence of pandemic-related triggers. Of the 414 patients seen in the emergency department, 129 were admitted to the hospital’s UHB. Of these, 86.0% tested negative on PCR for COVID-19; 20.9% of the admitted patients consulted for pandemic-related reasons; 14.7% of the analyzed subjects presented with delusions centered on the Corona virus. The results of the study revealed that the delusional content of 38.5% of the patients was influenced by COVID-19 and was the cause of 46.2% of the consultations requested. In the months that followed, the data showed a slight decline in consultations and delusions centered on the virus, due to the slow acclimatization and creation, in the more resilient subjects, of a new normality within which COVID-19 was incorporated. The data show that the peak in diagnoses of brief psychotic disorder and mutations in delusional characterizations occurred ponderously during the initial weeks of the pandemic; in the initial weeks, COVID-19 was the focus of every consultation and delirium in 100% of the cases analyzed. Thereafter, a slow decline occurred. The data show that in the second and third month the COVID-19 pandemic was a trigger in 17.6% and 11.5% of the consultations and the central factor in the delusions in 13.7% and 3.8% of the cases analyzed.

In this study, several clinical cases were examined, allowing the results to be seen and how COVID-19 was incorporated into the delusions. In the course of our systematic literature search, it was highlighted that the COVID-19 virus, in addition to affecting the respiratory system (general hypoxia), can also affect cerebral areas of the central nervous system inducing neuro-inflammation caused by an increase in pro-inflammatory cytokines. It was noted that in conjunction with respiratory symptoms, subjects infected with the COVID-19 virus exhibited delusional symptoms [28]. These conditions appear to contribute to the onset and/or worsening of psychiatric and neurological disorders. Evidence is reported that exposure to the virus, associated medical treatment and psychosocial distress are associated with the onset of psychosis. Psychiatric symptoms are conditioned not only by biological and medical conditions, but also by high stress factors such as the social profile outlined by the COVID-19 pandemic. It can be argued that the increase in diagnoses of schizophrenia spectrum disorder have been caused by the COVID-19 virus from both a medical and a social point of view. The link between the pandemic and the increase in diagnoses of psychotic disorders has already been highlighted in the past. Menninger conducted a study of 100 patients with influenza-associated neuropsychiatric sequelae, observing that 25 subjects presented with dementia praecox and 23 with psychosis, highlighting the link between the Spanish pandemic and psychotic disorders in 1918 (1918, cited by Brown et al., 2020) [5]. This was confirmed by a study conducted in China in 2003, which showed that SARS also induced an increase in psychosis. Among SARS survivors treated in the United Christian Hospital 30 months after the SARS outbreak in China, it was shown that the cumulative incidence of psychiatric disorders was 58.9%; among these, the incidence of post-SARS psychosis was 4.4%. The phenomenon of “influenza psychosis” has been documented during several pandemics including Ebola, MERS and swine flu [29]. In agreement with these findings, Hu et al. (2020) observed a 25% increase in schizophrenia diagnoses, compared to previous years, in areas where COVID-19 was prevalent [5]. Furthermore, through a review carried out on the subject by Professor Caponnetto et al. (2021) [7], we were able to observe how subjects with schizophrenia spectrum disorder, specifically diagnosed with schizophrenia, responded to the COVID-19 contagion quarantine, highlighting how the most environmentally flexible symptom is delirium. This systematic review examines various research approaches investigating the impact that social distancing had on subjects with schizophrenia disorder, focusing more on psychotic manifestations. Among the various studies is the study conducted by Jun Ma et al. (2020), which included an experimental group (subjected to social isolation following contact with a patient infected with the virus) and a control group, both consisting of a total of 30 patients from the Wuhan Health Centre with schizophrenia disorder. The results show that there were changes in the levels of stress, anxiety and depression in the experimental group compared to the control group but show that there are no changes in symptoms related to schizophrenia. This result may be related to the nature of the disorder [30].

## 5. Conclusions

The literature review we conducted allowed us to confirm our initial hypotheses by highlighting that the COVID-19 pandemic led to a mutation of delusional characterizations in individuals with schizophrenia spectrum disorder due to the radical changes that occurred in the social context. In fact, as evidenced by the literature on the subject, the delusional content, especially during the first weeks of the virus spread, focused mainly on topics related to the COVID-19 virus.

It was also observed that there was a spike in diagnoses of schizophrenia spectrum disorder caused by the enormous perceived psychosocial stress, specifically a weighty increase in diagnoses of brief psychotic disorder. Thus, it can be concluded, highlighting the veracity of our research thesis, that there has been a change in the pathology of delirium, which centered on COVID-19, in subjects with schizophrenia spectrum disorder and a weighty increase in diagnoses of schizophrenia spectrum disorder (specifically an increase in DPB) caused by the enormous perceived psychosocial stress. Furthermore, it has been shown that the overabundance of information (a phenomenon termed infodemia) caused by the mass media positively correlates with the development of conspiratorial thinking and the onset of delusions.

Several authors agree that the curve may tend to increase in the near future as a consequence of exposure to the COVID-19 virus. Future research should be directed at observing the changes that this period will induce in the long term and, in fact, several authors agree that the diagnosis curve for schizophrenia spectrum disorders will tend to increase as a consequence of exposure to stressors and the COVID-19 virus infection.

## Figures and Tables

**Table 1:** Cochrane risk of bias tool for randomized trials, version 2 (RoB 2).

Sections	Randomization Process	Deviations from Intended Interventions	Missing Outcome Data	Measurement of the Outcome	Selection of the Reported Result	Overall
Section 3.1	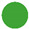	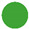	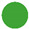	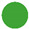	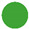	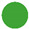
Section 3.2	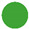	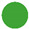	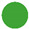	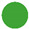	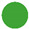	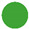
Section 3.3	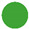	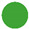	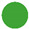	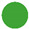	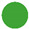	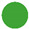
Section 3.4	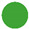	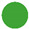	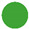	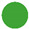	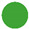	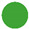
Section 3.5	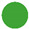	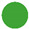	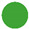	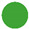	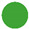	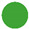
Section 3.6	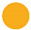	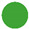	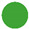	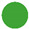	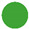	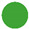
Section 3.7	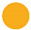	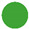	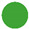	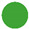	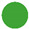	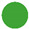
Section 3.8	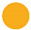	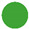	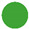	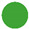	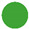	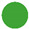
Section 3.9	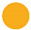	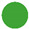	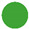	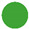	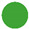	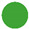
Section 3.10	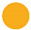	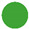	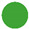	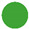	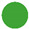	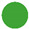
Section 3.11	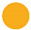	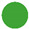	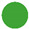	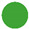	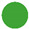	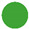
Section 3.12	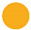	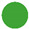	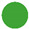	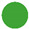	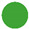	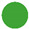
Section 3.13	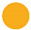	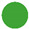	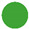	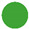	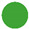	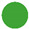
Section 3.14	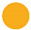	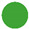	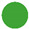	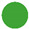	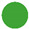	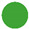

**Legend:** 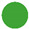 Low Risk, 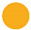 Some Concerns, 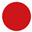 High Risk.

**Table 2:** Data Extraction.

Authors/Refs.	Year	Title	Nation	Type of Study	Sample	Measures	Results
Pérez-Balaguer, A. et al. [8]	2021	Does the COVID-19 pandemic affect the delusional content of patients with psychosis?	Spain	Observational, cross-sectional and retrospective study	414 subjects out of an initial sample of 430	Interviews	COVID-19 influenced the delusional content in 38.5% of patients and was the direct trigger in 46.2% of cases (during the first month).
Lopes, B. et al. [9]	2020	Paranoia, hallucinations and compulsive buying during the first phase of the outbreak of COVID-19 in the UK	United Kingdom	Preliminary experimentation	361 subjects	3 political trust indexes tapping Trust in Politicians, Trust in Congress, and Trust in the Political System (Mutz and Reeves, 2005) The Lubben Social Network Scale (Lubben et al., 2006) The Fear of COVID-19 Scale (Ahorsu et al., 2020) The Paranoia Checklist (Freeman et al., 2005) Launay and Slade’s predisposition to Hallucinations Scale (Launay and Slade, 1981) The compulsive buying behaviour scale (Edwards, 1993)	It has been highlighted that exposure to conspiracy theories and COVID-19-related information overload correlates positively with feelings of political distrust, paranoia and hallucinatory experiences.
Valdés-Florido, M.J. et al. [10]	2021	Clinical characterisation of short psychotic disorders triggered by pandemic COVID-19	Germany	Multicentre observational	33 subjects	Clinical profile observation, clinical interviews	In 57.6% of the cases, the delusional content was related to COVID-19. Half of the subjects experienced symptoms of first- order schizophrenia and about 24.2% symptoms of suicide.
D’Agostino, A. et al. [11]	2020	Short Psychotic Disorder during the national lockdown in Italy: an emerging clinical phenomenon of the coronavirus pandemic	Italy	Clinical observational	6 clinical cases	The Brief Psychiatric Rating Scale (BPRS), the Paykel interview, the Structured Clinical Interview for the DSM (SCID-II)	Change in delirium pathobiology (COVID-19 incorporated) and increase in schizophrenia spectrum disorders
Seifert, J. et al. [12]	2021	Peripatetic psychiatric emergencies: impact of COVID-19 pandemic on patients second diagnostic subgroup	Germany	Clinical observational	75 patients	Clinical profile observation, clinical interviews	Delusions centred on the COVID-19 virus
Behere, P.B. et al. [13]	2021	Has psychopatholog y in Indian psychiatric patients changed following the COVID-19 pandemic?	India	Clinical observational	Clinical cases	Clinical profile observation, clinical interviews	The pandemic induced the development of psychosis and how the content of the delusions incorporated COVID-19.
Finatti, F. et al. [14]	2020	Psychosis in quarantine patients related to COVID-19: a case series	Italy	Clinical observational	Clinical cases	Clinical profile observation, clinical interviews	The change in delirium pathobiology (COVID-19 incorporated) is highlighted
Sunbul, E.A., et al. [4]	2021	Brief psychotic disorder during the COVID-19 pandemic: a case series	India	Clinical observational	Clinical cases	Clinical profile observation, clinical interviews	The pandemic induced the development of psychosis and how the content of the delusions incorporated COVID-19.
Smith, C.M. et al. [15]	2020	Short psychotic disorder associated with COVID-19		Clinical observational		Clinical profile observation, clinical interviews	The pandemic induced the development of psychosis and how the content of the delusions incorporated COVID-19.
Shanbour, A. et al. [16]	2020	Psychosis and lymphodukaemi a isolation resulting in first hospitalisation during the COVID-19 pandemic: a case series		Clinical observational		Clinical profile observation, clinical interviews	The pandemic induced the development of psychosis and how the content of the delusions incorporated COVID-19.
Huarcaya-Victoria, J. et al. [2]	2020	Psychosis in a patient with anxiety related COVID-19: a case report		Clinical observational		Clinical profile observation, clinical interviews	Relationship between coronaphobia and the onset of psychotic symptoms.
Fischer, M. et al. [3]	2020	COVID-19 paranoia in a patient with schizophrenic psychosis		Clinical observational	Clinical case reports	Clinical profile observation, clinical interviews	The pandemic induced the development of psychosis and how the content of the delusions incorporated COVID-19.
Nava, R. et al. [17]	2020	Blockage and psychosis: a paranoid delusion		Clinical observational		Clinical profile observation, clinical interviews	The pandemic induced the development of psychosis and how the content of the delusions incorporated COVID-19.
Grover, S. et al. [18]	2021	Fear of spreading COVID-19 infection in a woman with psychotic illness leading to suicide and homicide attempts: a case report		Clinical observational		Clinical profile observation, clinical interviews	The fear of spreading and contracting the COVID-19 virus led to the exacerbation of related psychotic symptoms.

## Data Availability

Not applicable.

## References

[1] American Psychiatric Association (2013). Diagnostic and Statistical Manual of Mental Disorders.

[2] Huarcaya-Victoria J., Herrera D., Castillo C. (2020). Psychosis in a patient with anxiety related to COVID-19: A case report. Psychiatry Res..

[3] Fischer M., Coogan A.N., Faltraco F., Thome J. (2020). COVID-19 paranoia in a patient suffering from schizophrenic psychosis—A case report. Psychiatry Res..

[4] Sunbul E.A., Cavusoglu E.C., Gulec H. (2021). Brief psychotic disorder during COVID-19 pandemic: A case series. Indian J. Psychiatry.

[5] Hu Z., Yang Z., Li Q., Zhang A. (2020). The COVID-19 Infodemic: Infodemiology Study Analyzing Stigmatizing Search Terms. J. Med. Internet Res..

[6] Brown E., Gray R., Lo Monaco S., O’Donoghue B., Nelson B., Thompson A., Francey S., McGorry P. (2020). The potential impact of COVID-19 on psychosis: A rapid review of contemporary epidemic and pandemic research. Schizophr. Res..

[7] Caponnetto P., Benenati A., Maglia M.G. (2021). Psychopathological Impact and Resilient Scenarios in Inpatients with Schizophrenia Spectrum Disorders Related to COVID Physical Distancing Policies: A Systematic Review. Behav. Sci..

[8] Pérez-Balaguer A., Sanz-Aranguez-Ávila B., Gil-Benito E., Solari-Heresmann L.M., Sol-Calderón P.D., Gayubo-Moreo L., Arce-Cordón R. (2021). ¿La pandemia de COVID-19 condiciona el contenido delirante de los pacientes con psicosis? Un estudio observacional [Does the COVID-19 pandemic condition the delusional content of patients with psychosis? An observational study]. Rev. Colomb. Psiquiatr..

[9] Lopes B., Bortolon C., Jaspal R. (2020). Paranoia, hallucinations and compulsive buying during the early phase of the COVID-19 outbreak in the United Kingdom: A preliminary experimental study. Psychiatry Res..

[10] Valdés-Florido M.J., López-Díaz Á., Palermo-Zeballos F.J., Garrido-Torres N., Álvarez-Gil P., Martínez-Molina I., Martín-Gil V.E., Ruiz-Ruiz E., Mota-Molina M., Algarín-Moriana M.P. (2021). Clinical characterization of brief psychotic disorders triggered by the COVID-19 pandemic: A multicenter observational study. Eur. Arch. Psychiatry Clin. Neurosci..

[11] D’Agostino A., D’Angelo S., Giordano B., Cigognini A.C., Chirico M.L., Redaelli C., Gambini O. (2020). Brief Psychotic Disorder during the National Lockdown in Italy: An Emerging Clinical Phenomenon of the COVID-19 Pandemic. Schizophr. Bull..

[12] Seifert J., Meissner C., Birkenstock A., Bleich S., Toto S., Ihlefeld C., Zindler T. (2021). Peripandemic psychiatric emergencies: Impact of the COVID-19 pandemic on patients according to diagnostic subgroup. Eur. Arch. Psychiatry Clin. Neurosci..

[13] Behere P.B., Behere A.P., Chowdhury D. (2021). Did psychopathology in Indian psychiatric patients change following the COVID-19 pandemic?. Indian J. Psychiatry.

[14] Finatti F., Pigato G., Pavan C., Toffanin T., Favaro A. (2020). Psychosis in Patients in COVID-19-Related Quarantine: A Case Series. Prim. Care Companion CNS Disord..

[15] Smith C.M., Komisar J.R., Mourad A., Kincaid B.R. (2020). COVID-19-associated brief psychotic disorder. BMJ Case Rep..

[16] Shanbour A., Khalid Z., Fana M. (2020). Psychosis and Infodemic Isolation Resulting in First Inpatient Hospitalization During the COVID-19 Pandemic A Case Series. Prim. Care Companion CNS Disord..

[17] Nava R., Castiglioni M., Don P.W., Di Brita C., Colmegna F., Clerici M. (2020). Lockdown and Psychosis: A Paranoid Delusion. Prim. Care Companion CNS Disord..

[18] Grover S., Suman A., Naskar C., Jagota G., Sahoo S., Mehra A. (2021). Fear of spreading COVID-19 infection in a female with psychotic illness leading to suicidal and homicidal attempt: A case report. Asian J. Psychiatry.

[19] Mutz D., Reeves B. (2005). The New Videomalaise: Effects of Televised Incivility on Political Trust. Am. Political Sci. Rev..

[20] Lubben J., Blozik E., Gillmann G., Iliffe S., von Renteln Kruse W., Beck J.C., Stuck A.E. (2006). Performance of an Abbreviated Version of the Lubben Social Network Scale among Three European Community-Dwelling Older Adult Populations. Gerontologist.

[21] Ahorsu D.K., Lin C.Y., Imani V., Saffari M., Griffiths M.D., Pakpour A.H. (2020). The Fear of COVID-19 Scale: Development and Initial Validation. Int. J. Ment. Health Addict..

[22] Freeman D., Garety P.A., Bebbington P.E., Smith B., Rollinson R., Fowler D., Kuipers E., Ray K., Dunn G. (2005). Psychological investigation of the structure of paranoia in a non-clinical population. Br. J. Psychiatry J. Ment. Sci..

[23] Launay G., Slade P.D. (1981). The measurement of hallucinatory predisposition in male and female prisoners. Personal. Individ. Differ..

[24] Edwards E.A. (1993). Edwards Compulsive Buying Scale.

[25] Ferrando S.J., Klepacz L., Lynch S., Tavakkoli M., Dornbush R., Baharani R., Smolin Y., Bartell A. (2020). COVID-19 Psychosis: A Potential New Neuropsychiatric Condition Triggered by Novel Coronavirus Infection and the Inflammatory Response?. Psychosom..

[26] Sallam M., Dababseh D., Yaseen A., Al-Haidar A., Taim D., Eid H., Ababneh N.A., Bakri F.G., Mahafzah A. (2020). COVID-19 misinformation: Mere harmless delusions or much more? A knowledge and attitude cross-sectional study among the general public residing in Jordan. PLoS ONE.

[27] Gu F., Wu Y., Hu X., Guo J., Yang X., Zhao X. (2021). The Role of Conspiracy Theories in the Spread of COVID-19 across the United States. Int. J. Environ. Res. Public Health.

[28] Zhand N., Joober R. (2021). Implications of the COVID-19 pandemic for patients with schizophrenia spectrum disorders: Narrative review. BJPsych Open.

[29] Kepinska A., Plana-Ripoll O., Vernon A.C., Yolken R., Murray R.M., Pollak T.A. (2020). Schizophrenia and influenza at the centenary of the 1918-1919 spanish influenza pandemic: Mechanism of psychosis risk. Front. Psychiatry.

[30] Ma J., Jiang T., Huang H., Li R., Zhang L., Liu L., Liu X. (2021). Mental Symptoms and Stress of Hospitalized Schizophrenia Patients with 2019 Novel Coronavirus Disease: An Observation Study. Front. Psychiatry.

